# Usability Test of Exercise Games Designed for Rehabilitation of Elderly Patients After Hip Replacement Surgery: Pilot Study

**DOI:** 10.2196/games.7969

**Published:** 2017-10-12

**Authors:** Yun Ling, Louis P Ter Meer, Zerrin Yumak, Remco C Veltkamp

**Affiliations:** ^1^ Utrecht University Utrecht Netherlands; ^2^ Erasmus School of Health Policy and Management Rotterdam Netherlands

**Keywords:** rehabilitation exercise, computer games, hip replacement, elderly, physical therapists

## Abstract

**Background:**

Patients who receive rehabilitation after hip replacement surgery are shown to have increased muscle strength and better functional performance. However, traditional physiotherapy is often tedious and leads to poor adherence. Exercise games, provide ways for increasing the engagement of elderly patients and increase the uptake of rehabilitation exercises.

**Objective:**

The objective of this study was to evaluate Fietsgame (Dutch for *cycling game*), which translates existing rehabilitation exercises into fun exercise games. The system connects exercise games with a patient’s personal record and a therapist interface by an Internet of Things server. Thus, both the patient and physiotherapist can monitor the patient’s medical status.

**Methods:**

This paper describes a pilot study that evaluates the usability of the Fietsgame. The study was conducted in a rehabilitation center with 9 participants, including 2 physiotherapists and 7 patients. The patients were asked to play 6 exercise games, each lasting about 5 min, under the guidance of a physiotherapist. The mean age of the patients was 74.57 years (standard deviation [SD] 8.28); all the patients were in the recovery process after hip surgery. Surveys were developed to quantitatively measure the usability factors, including presence, enjoyment, pain, exertion, and technology acceptance. Comments on advantages and suggested improvements of our game system provided by the physiotherapists and patients were summarized and their implications were discussed.

**Results:**

The results showed that after successfully playing the games, 75% to 100% of the patients experienced high levels of enjoyment in all the games except the squats game. Patients reported the highest level of exertion in squats when compared with other exercise games. Lunges resulted in the highest dropout rate (43%) due to interference with the Kinect v2 from support chairs. All the patients (100%) found the game system useful and easy to use, felt that it would be a useful tool in their further rehabilitation, and expressed that they would like to use the game in the future. The therapists indicated that the exercise games highly meet the criteria of motor rehabilitation, and they intend to continue using the game as part of their rehabilitation treatment of patients. Comments from the patients and physiotherapists suggest that real-time corrective feedback when patients perform the exercises wrongly and a more personalized user interface with options for increasing or decreasing cognitive load are needed.

**Conclusions:**

The results suggest that Fietsgame can be used as an alternative tool to traditional motor rehabilitation for patients with hip surgery. Lunges and squats are found to be more beneficial for patients who have relatively better balance skills. A follow-up randomized controlled study will be conducted to test the effectiveness of the Fietsgame to investigate how motivating it is over a longer period of time.

## Introduction

### Background

Elderly people consume a large part of the health care and social services, especially in developed countries [1]. Hip fracture is considered a major problem for elderly people because of its high incidence [2,3] and the related high mortality and morbidity [4,5], and as a consequence the decreased quality of life visible in reduced physical movement [6], and finally the increased costs of health care involved in the treatment of patients [1,2]. The most commonly used treatment method is surgery, which helps patients recover more quickly [7]. Besides, fracture hip replacement is a treatment for patients with abrasion of the hip joints to reduce pain and increase mobility. Establishing rehabilitation programs after hip surgery improves the quality of life of elderly patients [6]. Earlier studies with patients who received outpatient rehabilitation showed increased strength and better functional performance such as self-care ability and mobility after 3 months and 1 year, respectively, compared with those without rehabilitation [5,8]. However, conventional physiotherapy is often experienced as boring by the patient, leading to poor adherence [9].

Due to the engaging, entertaining, and thus motivating properties of exercise games, gaming has been proposed as a valuable instrument to encourage patients’ participation in rehabilitation and improve patients’ adherence to therapy programs [10]. For example, Pichierri et al [11] and Uzor and Baillie [12] showed better adherence in exercise gaming groups than in the controlled conventional groups. Furthermore, playing an exercise game can be used to distract patients’ attention from pain resulting from their surgery or movement, and it thus contributes to the patients’ motivation to use exercise games [13,14].

Exercise games have shown equal or superior effectiveness compared with conventional physiotherapy in rehabilitation in patients over 16 years of age [15,16]. A meta-analysis suggests that exercise games are equally effective to improve balance, when considering the aspects of balance and walking speed [15]. Warburton et al [16] showed that, because of improved engagement of the patient, cycling exercise games result in significant improvements in physical fitness, including muscular strength and flexibility, compared with conventional cycling exercise training.

Compared with traditional rehabilitation, exercise games allow for task-specific exercises to be delivered at different difficulty levels. This allows the patient to start at an appropriate level and then proceed, based on a set of goals, with a gradual progression of difficulty. However, according to Skjaeret et al [10], the majority of the studies used commercially available gaming technologies such as the Nintendo Wii game console, Sony PlayStation II, X-Box360, and Dance Dance Revolution. These commercial games are originally designed for entertainment, targeted at younger people, and not based on exercise principles. Commercial games are also too difficult and not engaging enough for elderly. Therefore, effective exercise games that are specific to the needs of the elderly are needed.

Achieving the goal of rehabilitation after hip surgery requires accurate and appropriate tracking and feedback. Therefore, we developed Fietsgame using Microsoft Kinect as an off-the-shelf three-dimensional (3D) depth camera. Kinect v2 offers marker-free full-body tracking on a conventional personal computer (PC). It has a wide field- of-view to provide full-body control of animated virtual characters. This allows the virtual character on the screen to mirror the movements of the user in real time. Earlier studies concluded that Kinect v2 has the potential to be used as a reliable and valid clinical measurement tool [17-19]. Hence, the physiotherapists can set the range of knee angle and hip angle as they usually do in the traditional rehabilitation training when they use the exercise games.

### Objectives

To the best of our knowledge, only a few Kinect games offer exercises with full-body animated virtual characters and configurable level of difficulties, which are required for rehabilitation after hip surgery. Earlier studies using Kinect to design rehabilitation exercise games either focus on rehabilitation of the upper body [20-23] or use simple balance training exercises [24-27]. These games are lacking the variety of exercises with a very limited range of difficulty levels. We, therefore, have developed a series of immersive and motivating exercise games with real-time feedback and a configurable wide range of difficulty levels.

In general, applications supporting the management of illnesses or providing assistance in daily living activities for the elderly showed good usability and high acceptance [28,29]. For example, Arnhold et al [28] showed that applications for elderly diabetes patients have moderate to good usability. Hossain and Ahmed [29] found that elderly participants interacted with the virtual caregiver easily and were highly satisfied with its assistance during their daily activities. Exercise games are designed to elicit motivation for rehabilitation training. Studies testing the usability of exercise games among the elderly population showed that the games that were specifically designed for the elderly were positively evaluated by the elderly participants with respect to their usability, user acceptance, enjoyment, and its rehabilitation effect [30-32].

In this pilot study, we were interested in getting an insight into the point of view from the physiotherapists, in particular whether the exercise games satisfy the nature of a motor rehabilitation program for elderly patients after hip surgery (Research question 1) and whether they have the intention to use the exercise games to treat the patients in the future (Research question 2). Furthermore, we investigated whether the patients experienced a high level of presence and enjoyment and an expected level of exertion and pain (Research question 3), and whether they found the games easy to use and wanted to continue using the exercise games to do further rehabilitation (Research question 4).

## Methods

### Participants

In total, 2 physiotherapists (a male aged 31 years and a female aged 29 years) and 7 patients (5 females and 2 males) with age range of 60 to 82 years (mean 74.57, SD 8.28) from Aafje Rehabilitation Center in Rotterdam, The Netherlands, participated in this study. The patients were recovering from hip joint replacement (hip arthroplasty) or (unipolar) short-stem hemiarthroplasty surgery. The inclusion criteria of the patients were that they should be capable of performing the exercises and understanding the instructions of the exercise game. Patients with acute illness in the past 3 weeks, with mental disorders, or with poor visual acuity (not capable of seeing the visual features on the TV screen) were excluded.

All participants provided written informed consent before their participation in the experiment. After completing the experiment and answering the questions, they received a compensation gift. The exercise games imposed the same risk as a regular therapy session, because the patients performed the same exercises as part of their normal treatment. Whenever the patient was playing the game, a physiotherapist was always present. The load of the exercise games was comparable with the normal treatment for both the patients and the therapists, according to the physiotherapists. This study has been approved by the board of directors of the rehabilitation center of Aafje and the ethical committee of Utrecht University.

### The Fietsgame

The Fietsgame has been designed by a consortium of physiotherapists, game designers, researchers, and an information technology company with the goal of improving the rehabilitation process. The specific aim was to increase the mobility of the joints and surrounding soft tissues and to increase muscular strength as well as endurance. The system has the following two components: the exercise games and Community Care 360 (CC360) with a therapist control interface and the patient’s medical record. The exercise games and CC360 are connected by the Internet of Things (IoT) server from Consultants to Government and Industries (CGI) [33]. The games were run on a PC with Windows 10 software and displayed on a 48-inch TV. A Raspberry Pi device was used to connect the PC to the IoT platform.

**Figure 1:**
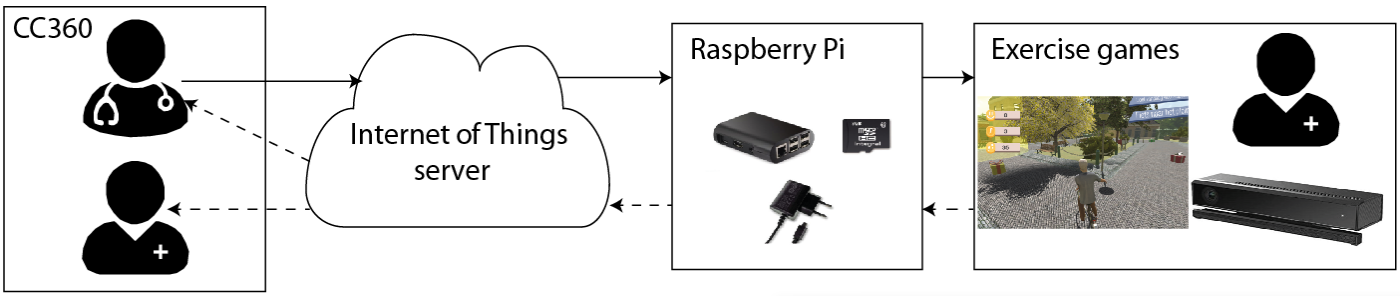
The Fietsgame system.

Figure 1 shows the architecture of the Fietsgame. The system works as follows: first, basic information such as the age, the date of intake, and a photo of the patient is fed into the patient’s medical record in CC360 and sent to the IoT server. Then, the patient logs into the system through a face recognition technology embedded in the exercise game using Kinect v2. We used face recognition to identify the user because of the following two reasons: first, it allows natural interaction with the system, with high recognition accuracy [34-37], and second, in the future, we can extend the exercise game system with emotion recognition using the camera [38-40].

After the exercise game recognizes the identity of the patient, assigned workout is automatically retrieved from the server using the Raspberry Pi. When the patient completes the exercise game, his or her workout data such as the number of exercises, knee or hip angles, and game scores are sent to the IoT platform and stored for further analysis. Both the patient and the physiotherapist can read the patient’s workout data through CC360.

### The Exercise Games

The purpose of hip rehabilitation is to reduce symptoms such as pain and inflammation and improve hip joint function approached through a systematic progression, depending on the patient’s present pathology and functional needs. The patients must understand the related precautions and the recommended progression for their individual situations. The physiotherapists advise a suitable exercise program by defining frequency, duration, and range of motion after considering the patient’s level of discomfort and physical status of the hip joints [41]. In our case, we created the exercise games based on the physiotherapists’ advice and requirements over a period of 4 years. The physiotherapists gave their suggestions and requirements to the design team regarding the exercises they need to have and what parameters the design team needs to configure in the rehabilitation program. Before the pilot test, we did 2 usability tests with 2 real patients and 2 physiotherapists to improve the usability issues of the earlier version of the exercise games. This paper presents the results of testing the beta version of the exercise games.

**Figure 2:**
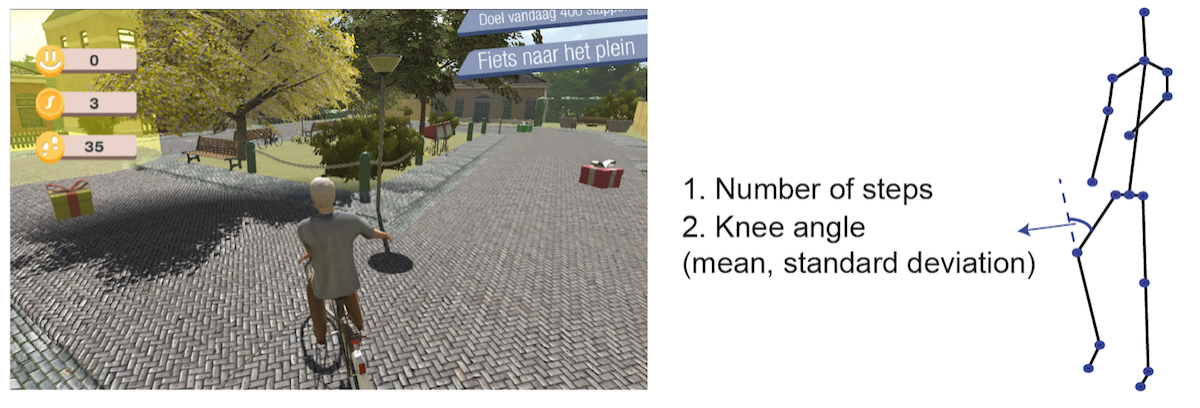
The cycling game—stepping; left: virtual environment, right: configurable variables.

**Figure 7:**
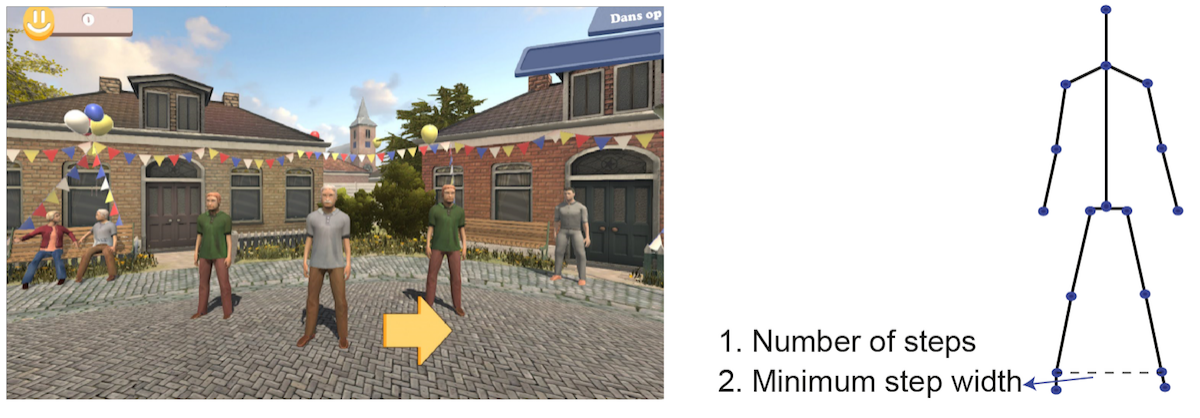
The dancing game—sidestepping; left: virtual environment, right: configurable variables.

**Figure 3:**
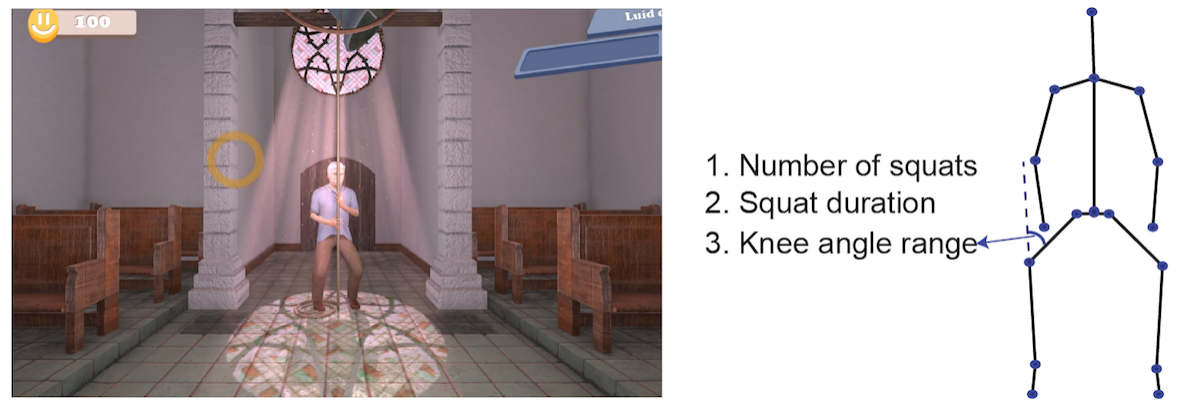
The ringing the bell game—squats; left: virtual environment, right: configurable variables.

**Figure 4:**
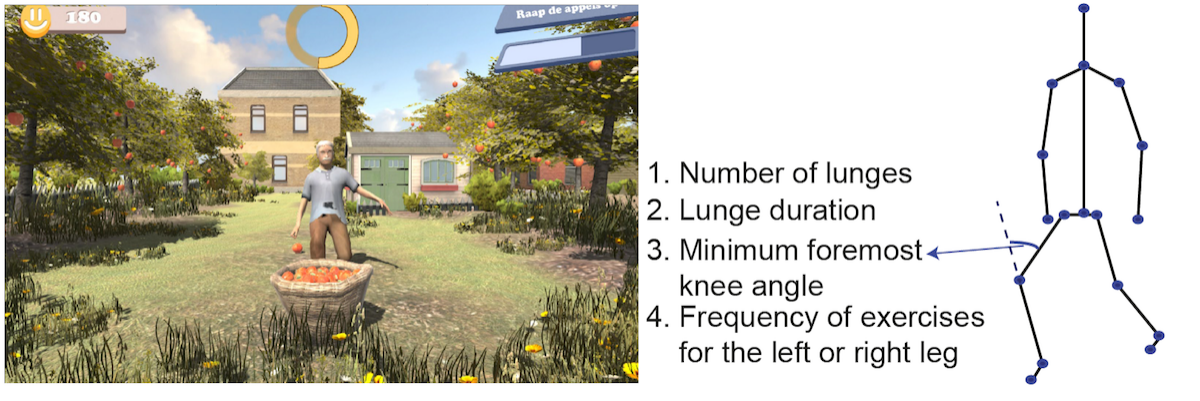
The apple picking game—lunges; left: virtual environment, right: configurable variables.

**Figure 5:**
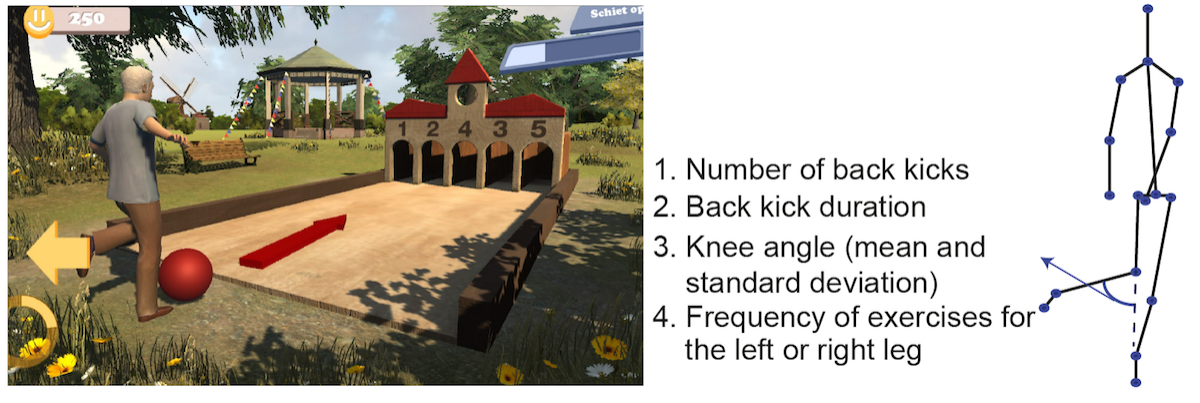
The football playing game—back kicks; left: virtual environment, right: configurable variables.

**Figure 6:**
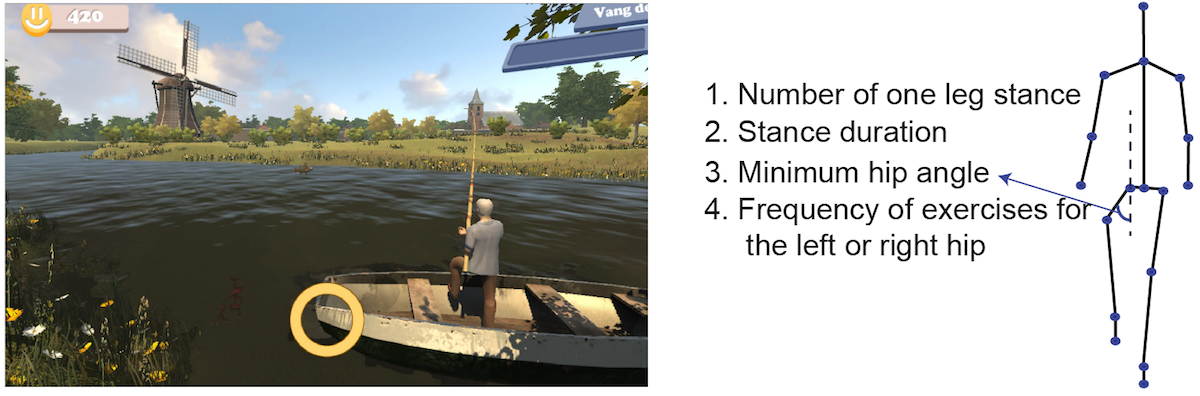
The fishing game—one leg stance; left: virtual environment, right: configurable variables.

The games are implemented using the Unity 3D game engine. There are 6 exercise games with 6 different balance exercises: cycling in a life-like virtual village for stepping, dancing under the spotlight with fellow dancers for sidestepping, ringing the bell in a church for squats, picking up apples for lunges, playing football for back kicks, and fishing on a boat for single leg stance (Figures 2-7). The participant’s avatar is presented from a third person perspective. The therapist can adapt the difficulty level of the exercise games according to the patient’s physical condition and level of discomfort. Possible configuration parameters for each game are shown in Figures 2-7. In addition to the final scores, the games consist of motivational elements such as awards and sounds. The interface of the game is in Dutch. In the following paragraphs, we summarize the play of each game.

#### Cycling

In the cycling game (Figure 2), the player is instructed to finish stepping exercises with the minimum required knee angle. When the player performs the exercises correctly, the bike goes forward smoothly. If not, the bike stops going forward. There are gift boxes and pedestrians on the road. An arrow indicates when the player comes to the end of the road. The player needs to put out his/her left (or right) hand to turn left (or right). If the player follows the direction of the arrow, she/he can get to the destination faster. The player can pick up the gift box to earn money by running over it and can wave to the pedestrians to earn social points. When the player waves at the pedestrians, the bell of the bike rings. The configurable variables in this game are the number of steps and required knee angle with mean and SD.

#### Dancing

The dancing game (Figure 3) works as follows: the player is expected to do the sidestepping exercises with the minimum required step width. The arrow in the interface indicates which side the player should take a step. When the exercise is performed successfully, the avatars in the virtual environment give feedback by dancing and clapping their hands. The avatars stay in the standing pose if the player does not perform the exercise correctly. The configurable variables are the number of steps and minimum step width.

#### Ringing the Bell

During the ringing the bell game (Figure 4), the player is expected to do squats with the required knee angle and duration as defined in the configuration file. When the squat pose is correct, the avatar mimics the player and a circle indicating the progress starts to fill with brighter colors to count for the duration of the squat. When the patient manages to stay in balance for the required duration of the squat, the circle is fully bright and the bell rings, indicating the accomplishment of the exercise. When the patient fails in doing the squat correctly, the progress circle does not fully turn to a bright color. Furthermore, the brightened part of the circle disappears if the player fails to hold the correct squat pose for the required duration and needs to start again. The configurable variables are the number of squats, squat duration, and knee angle range (minimum and maximum).

#### Apple Picking

The apple picking game (Figure 5) asks the player to perform lunges with a required minimum front knee angle. An apple falls from the left/right side of the avatar, and the player should step the mirrored left/right leg forward to perform lunges. The function of the yellow circle works similar to the one in the ringing the bell game. The avatar mimics the player’s movement, and the circle starts to fill with brighter colors to count for the duration of the lunge when the lunge pose is correct. When the patient manages to stay in balance for the required duration, the circle is fully bright and the apple is picked up and thrown into the basket. When the patient fails in doing the exercise correctly, the yellow circle does not start to fill with brighter colors to count the duration of holding the lunge. The brightened part of the circle disappears if the player fails to hold the correct pose for the required duration. The basket shown in the front of the avatar in the game is to show the award by playing lunges successfully. It has no other function in the game. The configurable variables are the number of lunges, lunge duration, minimum foremost knee angle, and the frequency of exercises for the left or right leg.

#### Football Playing

The goal of the football game (Figure 6) is to kick the ball to one of the 5 holes. At each turn, the number assigned to the holes is changed, and when the user aims at the hole with a higher number, he/she scores higher. The player raises one of the legs to prepare for a back kick and holds the leg in that position for the duration defined in the settings. The user turns his/her upper body to left/right to aim for the hole while keeping the leg raised. An arrow indicates which leg should be used to do the back kicks. The real-time feedback function of the yellow circle is the same as the ones in the ringing the bell and the apple picking games. Once this duration of back kick is completed, feedback is provided by showing the avatar kicking the football out. No kicking actions of the avatar will be shown when the player does not perform the exercise correctly. The configurable variables are number of back kicks, back kick duration, knee angle (mean and SD), and the frequency of exercises for the left or right leg.

#### Fishing

The objective of the fishing game (Figure 7) is to perform single leg stance. The player is required to perform one-leg stance exercise with a configured minimum hip angle and holding time. A fish is caught and put into the boat when the player performs the exercise correctly in each turn. An arrow indicates which leg should do the one leg stance. The real-time feedback function of the yellow circle is the same as the ones in the ringing the bell, the apple picking, and the football playing games. No fish will be caught if the player fails in doing one leg stance. The configurable variables are the number of one leg stances, stance duration, minimum hip angle, and the frequency of exercises for the left or right hip.

### Community Care 360

**Figure 8:**
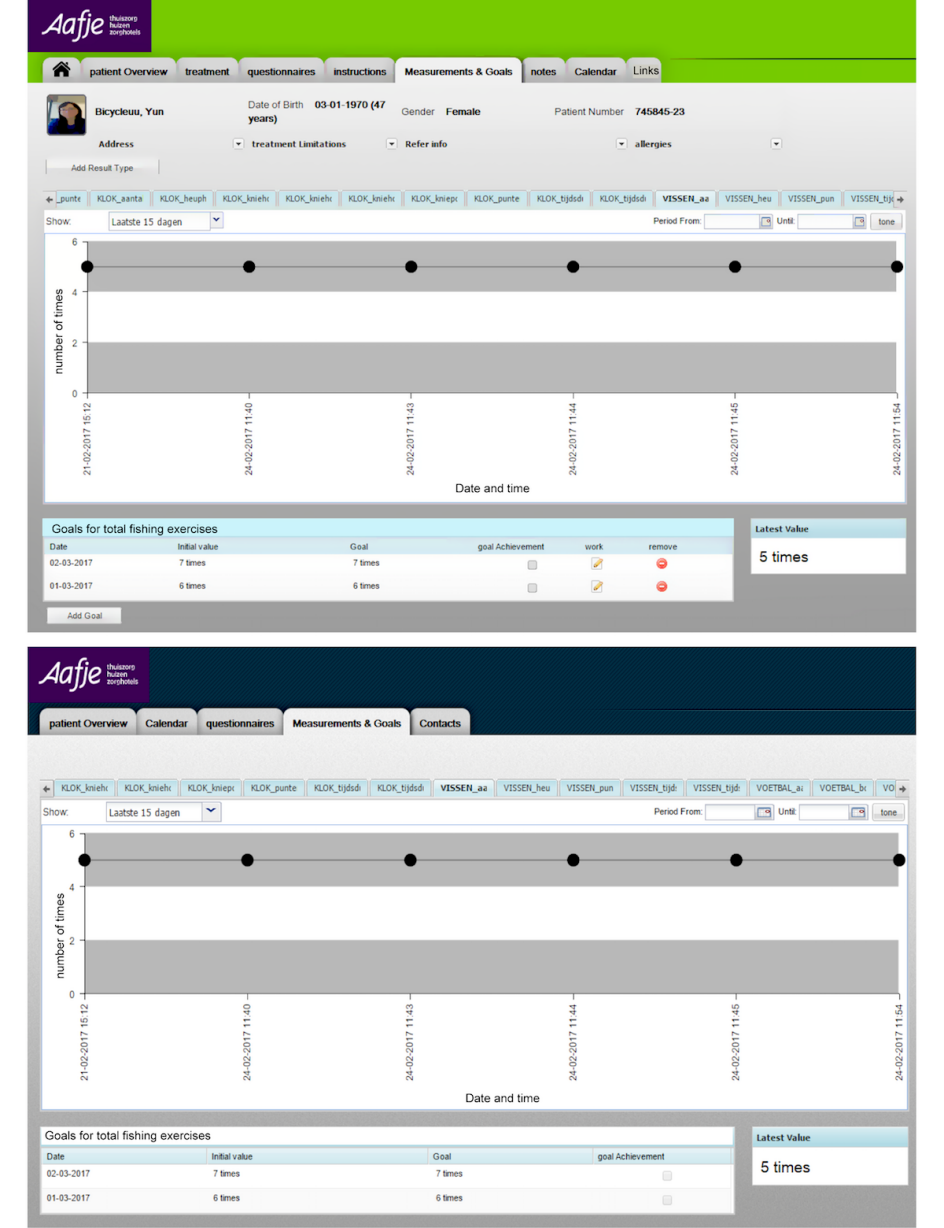
Top: the physiotherapist control interface; bottom: the patient interface showing patient’s medical record in CC360. The configuration parameter vissen_aantal means the number of fishing exercises.

CC360 is a patient-centric health platform that allows the patients, health care professionals, and other stakeholders to monitor and manage the patients’ health. CC360 provides applications for both the therapists and the patients. The configuration interface (Figure 8 top, originally in Dutch; see also Multimedia Appendix 1) allows the physiotherapists to set the goals of the game according to the patients’ conditions and the rehabilitation goals. The therapist can specify the treatment for each patient such as the required range of the knee and hip angles during the game, depending on the patient’s physical capabilities. Thus, the therapist application allows the physiotherapists to work more effectively, following whether a patient is following the treatment plan and being prepared for consultations. In the patient application (Figure 8 bottom, originally in Dutch; see also Multimedia Appendix 2), the patient can see the exercises assigned to him/her by his/her physiotherapist and play the exercise games by performing the required therapeutic movements. The game software assesses the performance of the patient by analyzing the data captured by Kinect v2. The final results, such as the number of successfully accomplished movements, knee angle, hip angle, and start and end time of the exercises, will be recorded and sent to the IoT platform and can be accessed through the therapist and patient applications in CC360. The exercises can also be assigned through a local configuration file on a PC when the patient receives no assignment from the physiotherapist through CC360.

### Measures

The measurements used in the experiment include psychometric tools, such as self-reported questionnaires, and objective behavioral measurements. Objective behavioral measures such as knee angle, step width, hip angle, and the number of successfully finished exercises were captured by Kinect v2 and sent to the IoT platform via the Raspberry Pi. The experiment was also video-recorded for further analysis of the comments of the therapists and the patients during the exercise game. Self-reported questionnaires were filled in by the therapists and were answered by the patients. The questionnaire for the patients was designed to measure the subjective feeling of presence, enjoyment, exertion, pain level, and technology acceptance, whereas the questionnaire for the physiotherapist was aimed to get an expert opinion on the usability of the game from the technology acceptance and rehabilitation point of view. More details about the questionnaires are given below, and the questionnaires for patients and physiotherapists are attached in Multimedia Appendices 3 and 4, respectively.

#### Self-Reported Questionnaires for the Patients

At the beginning of the experiment, patients were asked to fill in a questionnaire containing the following personal data: date of intake, the current number of daily exercise sessions, age, gender, mother tongue, gameplay experience, and social status. Visual acuity was measured using the Freiburg Visual Acuity Test at a distance of 3 m [42]. The experience of playing the exercise game was measured through standard questionnaires, including feelings of presence, enjoyment, exertion, pain level, and technology acceptance.

The concept of presence in virtual reality covers three aspects: spatial presence, social presence, and copresence [43]. In this study, we are interested in testing whether patients’ attention can be distracted by the real world. Therefore, spatial presence where patients’ feeling of being present in the virtual environment instead of being aware of the real world is important. Schubert et al [44] created an Igroup presence questionnaire (IPQ), which consists of 14 items rated on a 7-point Likert scale to measure spatial presence. The scores on the 14 IPQ items are mapped onto 3 subscales: spatial presence (the relation between the virtual reality and the physical real world), involvement (the awareness devoted to the virtual reality), and experienced realism (the sense of reality attributed to the virtual reality). It also includes one general item that assesses the general feeling of being in the virtual reality. To lower the burden of answering questions for the elderly, we measured presence using only the general item.

Enjoyment was tested by using a 1-item question on a 7-point Likert scale, “Do you find the exercise game interesting?” [45]. The Perceived Exertion Scale [46] was used as a measure of perceived exertion. It is a 15-point scale ranging from 6 (very light exertion) to 20 (very hard exertion). The Perceived Exertion Scale is widely used and has adequate reliability and validity. The perceived pain level was measured using the Visual Analogue Scale (VAS) [47]. The VAS contains 11 brief pain severity descriptions. Scores on the VAS ranged from 0 (no pain) to 10 (very severe pain).

The adapted Technology Acceptance Model (TAM) from Hu et al [48] was used in this study for measuring patients’ and physiotherapists’ intention to use the game system. It was suggested that ΤΑΜ was able to provide a reasonable depiction of physicians’ intention to use telemedicine technology [48]. TAM consists of 21 items with the following 4 subscales: perceived ease of use, perceived usefulness of the technology, attitude toward using the technology, and intention to use the technology. The participants’ responses were rated on a 7-point Likert scale from −3 (strongly disagree) to 3 (strongly agree).

#### Self-Reported Questionnaires for the Physiotherapists

At the beginning of the experiment, the physiotherapists were asked to fill in a questionnaire, which recorded their age, gender, mother tongue, education, and gameplay experience. The experience of using the exercise game was investigated through questionnaires, including criteria for rehabilitation of the exercise game [49] and the adapted TAM from Hu et al [48]. The questions in the TAM questionnaires were virtually the same for both the patients and the physiotherapists, but the terms used were rehabilitation and patient care for patients and physiotherapists, respectively.

Regarding the usability of the game for motor rehabilitation, we used a revised version of the design criteria for stroke rehabilitation programs for elderly users from Flores et al [49]. It includes the following five criteria:

1. Adaptability to the motor skill level of the patient. As motor impairments vary among patients and patients’ motor skills improve over time, the changeable level of difficulty in the exercise game is necessary.
2. Meaningful tasks. Tasks should be incorporated so that exercises in the game can be correlated with daily life activities.
3. Appropriate feedback for both the patient and the physiotherapist. The exercise game should provide real-time feedback on how well the patient is doing and how much she/he has been improving and provide encouraging feedback to stimulate the patient to adhere to the exercise game. Providing exercise record such as charting the history of patients’ exercise accomplishments can help the physiotherapist to better plan future therapy sessions.
4. Therapy appropriate range of motion. This refers to the extent the game demands the therapeutic motions needed for the rehabilitation program of patients after hip surgery.
5. Focus diverted from exercise. The game should be fun enough to divert patients’ attention from the exercises to the objectives of the gameplay.

Participants’ responses were rated on a 7-point Likert scale from −3 (strongly disagree) to 3 (strongly agree).

#### Qualitative Feedback

After playing each exercise game, all the participants were asked to give general feedback and comments on each game. At the end of the experiment, participants were asked to discuss their favorite and least favorite part of playing the exercise game in open questions.

### Procedure

To ensure high quality of recognition, we tested the exercise games in a controlled environment. To be more specific, the camera was set to track the closest person as long as possible, and only the player is within a distance of 2 to 3 m in front of the camera where the tracking accuracy is the best [50]. No objects or other persons are between the player and the Kinect v2 camera. There were 3 experimenters, 1 for the technical support of CC360 and making notes of the comments given by the patients and the physiotherapists, 1 for the technical support of the exercise game and also for making notes of the comments from the participants, and 1 for administrating the consent, questionnaires, and debriefing of the experiment.

After obtaining consent and basic information from the patients and the physiotherapists, participants were introduced to the exercise games, including the Kinect v2 sensor and CC360. One of the experimenters took a picture of the patient and uploaded it to the IoT platform for facial recognition to start the exercise games. The therapist then assigned the exercises according to the patient’s recovery status through the configuration file on a PC. The patients were asked to play 6 different exercise games, each lasting about 5 min. The physiotherapist was always in the same room as a guide for the patient and answered all the questions the patient asked during the game. Each participant was assessed individually during the session, which in total lasted about 60 min. Patient’s behavior and the voice of the experimenters and the physiotherapist were recorded by a laptop camera for later transcription.

Before each exercise game, the physiotherapist showed how to play the exercise game correctly and explained the instructions on the screen. Participants, if applicable, wore their prescription glasses during the experiment. All the patients used chairs to prevent falling. The chairs were placed on the left or right and behind the player. After each exercise game, the patients were asked to report their experienced level of presence, enjoyment, perceived exertion, and pain level by one of the experimenters and their reported scores were noted in the printed hard copy of questionnaire; the physiotherapists were asked to fill in a short questionnaire, which measures whether the exercise game meets the criteria for rehabilitation. Both the patient and the physiotherapist were asked to give a general feedback and comments on the game that the patient just played. Objective behavioral measures, including knee angle, step width, and hip angle, and the number of successfully finished exercises, were captured by Kinect v2 during the gameplay.

At the end of the experiment, both the patients and the therapists were asked to fill in the questionnaire for the TAM and to give their general comments about the exercise games. The patients were also asked whether they felt any discomfort before they left the room. They were requested to rest until they feel better when they experienced any discomfort. After completing the experiment, participants were debriefed and given a gift of 10 euros for their contribution. As CC360 is a widely used commercial product [33], we did not ask the patients or physiotherapists to use and evaluate CC360 itself.

### Data Analyses

All the behavioral data and self-reported scores of the questionnaires were analyzed with SPSS statistics package version 24. To answer our research questions, the measured data, including behavioral and self-reported data, were analyzed using descriptive statistics for all the 6 exercise games, and 2 trained researchers coded the qualitative feedback from patients and physiotherapists separately. Under the broad question, themes emerged from the coded data. The researchers discussed and refined the codes, that is, codes with similar meanings were grouped together, and the more frequently a code appeared, the more the theme was strengthened. We then analyzed the codes addressing gaming experience, game design, system operation, usefulness, and intention to use the exercise games.

## Results

### Patient Descriptions

A summary of demographic data and personal information of the patients is provided in Table 1. All the patients had their intake after hip surgery at Aafje Rehabilitation Centre between January and November in 2016. The pilot test was conducted in December 2016. Patients’ visual acuities were sufficient for playing the exercise games, which were rendered on a 48-inch TV placed at a distance of 3 m in front of them.

Median and interquartile ranges of workout assignments in the configuration from the guiding physiotherapist are provided in Table 2. Some of the configured variables such as the required knee angle in the cycling game were the same for all the patients. To keep the configuration of the knee angle more consistent between different exercise games, we need to change the mean and SD of the knee angle into minimum and maximum knee angles for the cycling game and the football playing game in the next version of the games.

**Figure 9:**
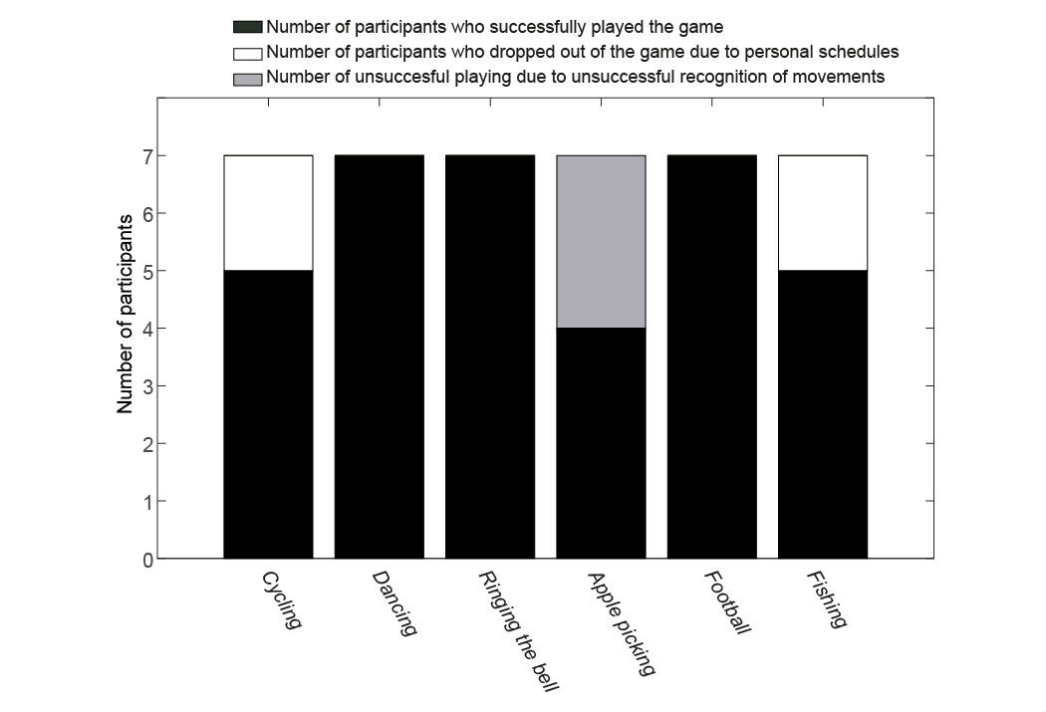
Number of participants who successfully played the game, who dropped out of the game, and who could not finish the assignments because of the unsuccessful recognition of their movements.

All the patients used chairs to keep balance during the exercises. Figure 9 shows the number of participants who successfully accomplished assignments from the physiotherapist, who dropped out because of personal reasons, and who could not finish the exercise because of recognition errors. Of the patients, 2 dropped out of cycling and fishing because of personal schedules, and 3 participants had to give up playing apple picking to do lunges because the exercise game could not recognize their movement even though they exercised correctly according to the guiding physiotherapist.

**Table 1:** Demographic and personal data of the patients.

Patient	Planned exercises per day	Age	Gender	Native language	Visual acuity	Sport	Frequency of playing computer games	Living status
1	2	70	Female	Dutch	0.69	Physio-training	Occasionally	Alone
2	3	82	Female	Dutch	0.48	Fitness and physio-training	Everyday	With partner
3	6	82	Male	Dutch	0.66	Physio-training	Never	With partner
4	3	60	Female	Dutch	0.73	Swimming, walking, and physio-training	Never	Alone
5	4	81	Female	Danish	0.64	Nordic walking and physio-training	Never	Alone
6	2	77	Female	Hungarian	0.53	Physio-training	Never	Alone
7	4	70	Male	Dutch	0.53	Coordinating football in the field and physio-training	Never	Alone

**Table 2:** Patients’ workout assignments in the exercise games (medians and interquartile ranges).

Games	Behavioral measurements	Median	Interquartile range
**Cycling**	Repeated number of steps	40	56
	Mean knee angle (degree)	45	N/A
	Knee angle standard deviation (degree)	10	N/A
**Dancing**	Repeated number of steps	10	N/A
	Minimum step width (cm)	30	10
**Ringing the bell**	Repeated number of squats	5	N/A
	Squats timer (second)	2	1
	Knee angle minimum (degree)	40	N/A
	Knee angle maximum (degree)	100	N/A
**Apple picking**	Repeated number of lunges	5	N/A
	Time to hold the lunges (second)	2	0
	Foremost minimum knee angle (degree)	30	0
**Football playing**	Repeated number of back kicks	10	N/A
	Standing timer (second)	3	0
	Mean knee angle (degree)	30	N/A
	Knee angle standard deviation (degree)	10	N/A
**Fishing**	Repeated number of one leg stance	10	N/A
	Standing timer (second)	3	1
	Minimum hip angle (degree)	80	20

### Patients’ Gaming Experience

**Figure 10:**
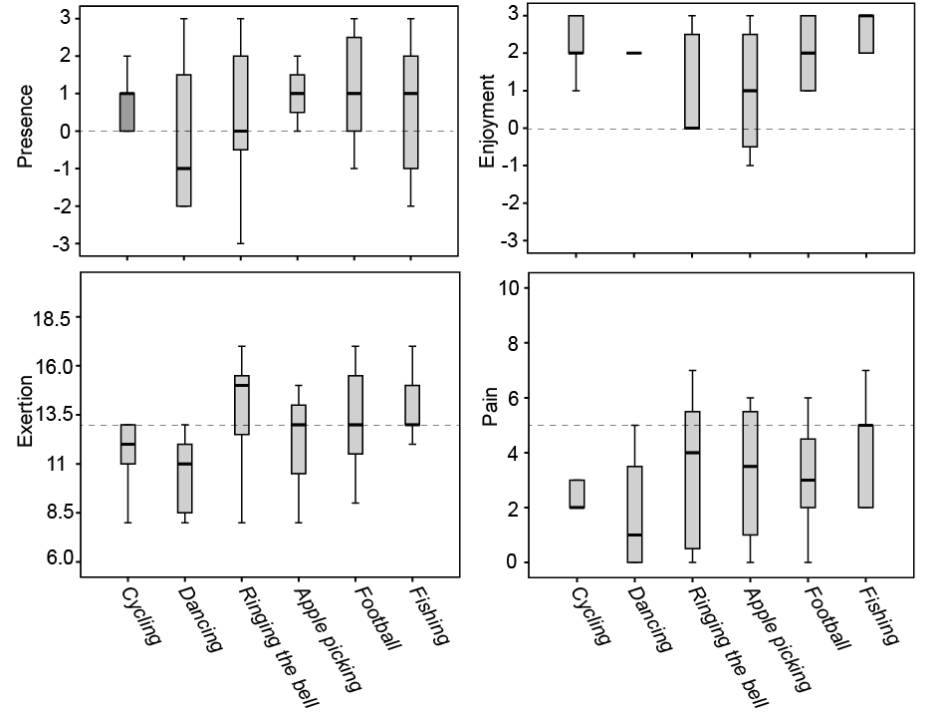
Medians and interquartile ranges of patients’ perceived feeling of presence, enjoyment, exertion, and pain for all the 6 exercise games. Scoring of presence and enjoyment ranged from −3 to 3, scoring of exertion ranged from 6 to 20, and scoring of pain ranged from 0 to 10. The horizontal line represents the median and the distance between the top, and the bottom of the bar represents the interquartile range.

Medians and interquartile ranges of patients’ perceived feelings of presence, enjoyment, exertion, and pain during each exercise game are shown in Figure 10. The score of each game was only from those participants who successfully accomplished the assignment as assigned by the physiotherapist in the exercise game. The majority of the participants reported high levels of presence (with presence score >0) in cycling (60%), apple picking (75%), football (71%), and fishing (60%) but a low level of presence in dancing (57% of the participants scored enjoyment below 0). Presence score for ringing the bell was evenly distributed over the score range. All the participants (100%) found cycling, dancing, and football enjoyable (with enjoyment score >0). Most of the participants found apple picking (75%) and fishing (80%) enjoyable to use, but only 43% of the participants rated ringing the bell enjoyable. All the participants (100%) experienced low to moderate exertion (exertion ≤13) in cycling and dancing. The majority of the participants also indicated low to moderate levels of exertion in apple picking (75%), football (57%), and fishing (60%), and a high level of exertion (exertion >13) in ringing the bell (57%). Most of the participants had low to moderate pain (score ≤5) while playing the exercise games such as cycling (80%), dancing (100%), ringing the bell (71%), apple picking (75%), football (86%), and fishing (80%). Very few participants reported a pain score above 5 during their exercises.

### Physiotherapists’ Evaluation of Game Design

The scores on the 5 items about whether the exercise games satisfy the nature of a motor rehabilitation program from 2 physiotherapists are shown in Table 3. Both physiotherapists expressed positive attitudes toward the exercise games on all the 5 items: (1) adaptability of the game to the motor skill level of patients, (2) providing meaningful tasks to promote quality of life, (3) giving appropriate feedback for both the patient and the physiotherapist to encourage adherence to the game and keep track of the patient’s recovery status, (4) staying within therapy-appropriate range of motion, and (5) diverting the patient’s consciousness from exercise toward game playing.

### Technology Acceptance Model

**Figure 11:**
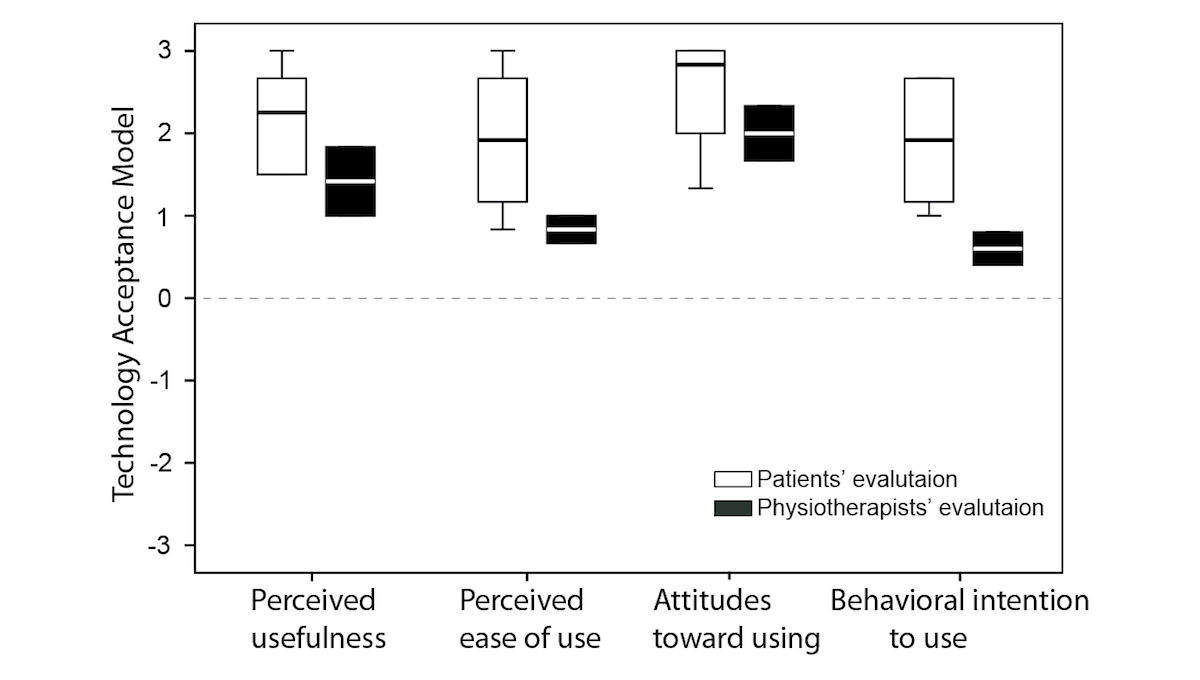
Medians and interquartile ranges of evaluation of Technology Acceptance Model (TAM) from both the patients and the physiotherapists. Scoring ranged from −3 to 3. The horizontal line represents the median, and the distance between the top and the bottom of the bar represents the interquartile range.

Figure 11 presents the medians and interquartile ranges of TAM items that were evaluated by both the patients and the physiotherapists. The exercise games were considered to be useful and easy to use. The participants expressed positive attitudes toward using the exercise games, as well as an intention to continue using the exercise games in their future rehabilitation or patient care.

**Table 3:** Evaluation of game design (scoring ranged from −3 to 3).

Game design evaluation			Cycling	Dancing	Ringing the bell	Apple picking	Football playing	Fishing
**Physiotherapist 1**								
		Adaptability	2	2	0	1	0	2
		Meaningful tasks	1	2	2	2	1	2
		Appropriate feedback	1	2	2	2	2	2
		Range of motion	2	2	2	2	2	2
		Diverted focus	2	2	2	2	2	2
**Physiotherapist 2**								
	Adaptability		2	2	1	2	2	2
	Meaningful tasks		2	2	1	3	2	3
	Appropriate feedback		2	2	1	2	2	3
	Range of motion		2	2	1	2	2	2
	Diverted focus		3	3	1	3	1	3

### Qualitative Feedback and Implications for Design Guidelines

Comments provided by the physiotherapists and the patients and their implications are presented in Table 4. In general, we received very positive feedback. The patients liked and enjoyed the games a lot, and sometimes they were genuinely excited. The game design, for example, the beautiful virtual environment, background music, and rewarding sound, brought in enjoyment to the patients. The patients can quickly exercise on their own in the exercise games. The players found the gameplay experience comparable with real physiotherapy and expressed intention to play again. Despite the overall positive comments on the *gaming experience*, *usefulness*, and *intention for future use*, we also encountered some issues relating to *game design* and *movement recognition by Kinect v2*. We discuss the main issues revealed from the negative comments and the corresponding implications for future work below. The requirements and implications hold for all the Kinect exercise games targeting the elderly population.

### Game Design

#### Cognitive Load

For some players, the exercise games were too complicated because of the requirement of engaging in multiple activities simultaneously. For example, in the cycling game, the patients sometimes need to do the stepping exercise, collect gift boxes and wave to the virtual pedestrians, and indicate directions occasionally at the same moment. Some patients found it difficult to follow the instruction arrow indicating which leg they should practice in the football playing game. However, most of the patients thought the game interface was rather easy to understand. Minimizing the amount of information presented on the screen might allow older patients with poor cognitive skills to perceive information. Hence, older patients can follow the instruction and commands more easily [28]. To tailor our exercise games to individual needs of cognitive challenges, picking gift boxes and waving to the other virtual humans should be optional in the cycling game. We believe that as the patients become more experienced with the games and their performances improve, the cognitive load of the games can be made more challenging by introducing levels. In this way, patients can be mentally challenged over a longer period, thus maintaining long-term exercise habits.

#### Lack of Real-Time Feedback on Wrong Movements

Some patients were unsure of what action should take place at a particular time. Providing helpful information and feedback at the appropriate time throughout the game will be beneficial [51]. For example, a real-time instruction such as how far away the patients’ knee angle is from the required knee angle should be given when the patient is performing the cycling game. How far the patients are away from achieving the minimum step width should be shown on the screen while playing the dancing game. Similarly, instructions such as how far away the patient’s knee/hip angles are from the required knee/hip angles and how much time is left for the required duration should be given when the patient is performing the rest of the games.

#### Mismatched Movements

Some patients got confused when the movement of the representing avatar did not match their movements when playing the dancing and football games. The patients were asked to do sidestepping without clapping hands when playing the dancing game, and they do not need to shoot the ball when doing back kick in the football game. Instructions showing that movements such as clapping hands and shooting the ball are not compulsory should be given at the beginning of the games to help the patients understand how the games actually worked.

#### Balance Skills

Patients at the beginning phase of their physiotherapy found some of the games more difficult. For example, it was difficult for them to do sidestepping in the dancing game. During the cycling game, waving to the avatars and indicating directions raising one of the hands were difficult for some of them because of impaired balance. Therefore, we suggest that the stepping exercise should not require the patient to go to the same side for more than once. Waving to the avatars can be optional in the game. Other ways for indicating directions such as turning the upper body or automatically changing directions should be configurable for patients with poor balance skills.

#### Indistinguishable Objects

Patients complained that all the fishes looked similar and they prefer varieties in the game content.

### Kinect Tracking

The Kinect did not properly recognize squats and lunges played by a few of the female patients with a wide blouse or obesity. Chairs, mirrors, and other objects in the environment sometimes interfered with the Kinect tracking. Of the participants, 3 had to quit playing the apple picking game as their lunges were not recognized. We observed that Kinect v2 could recognize lunges for patients with relatively normal mass level even with support chairs; however, for patients with obesity, it did not recognize their lunges. Furthermore, we found that Kinect v2 could not recognize movements of patients with a wide blouse or trousers; it worked better when they changed their clothes to relatively tight ones. Hence, we suggest that players wear relatively tight clothes when playing Kinect exercise games.

**Table 4:** Comments made by the physiotherapists, the patients, and the implications. Note that feedbacks from the physiotherapists are in *italic*.

Exercise games	Positive comments	Negative comments	Implications
Cycling	“I like this game a lot!”	*“It is difficult for the patients who are at the beginning phase of their physiotherapy to wave to other virtual humans or to put out a hand to indicate direction.”*	Picking gift boxes and waving to the other virtual humans should be optional in the game.
	“I like the beautiful village in the virtual environment.”	“The bike runs too fast and it made me dizzy.”	The speed of the bike should be configurable.
		“I tried not to run over the gift boxes on the street.”	Other ways for indicating directions such as turn the upper body should be configurable.
Dancing	“The game is nice. It is much better than the boring exercises we normally do.”	*“As the patients were using chairs to support balance, it was difficult to go to the same side for 2 or more side steps.”*	For patients who need support for balance, stepping exercise should not require the patient to go to the same side for more than once.
	“The music is good.”	“My movements did not match with the movements of the dancing avatars on the screen.”	Patients should be told that they could clap their hands if they want, but it is not required in the exercise.
	“I really looked forward to dancing and it was even better than I expected.”		
	“The game looks easier than it really is.”		
Ringing the bell	“It is a nice game.”	*“It is a difficult game; hence, patients’ pleasure is lost.”*	It is a difficult game for the patients, and it is more suitable for patients who have better balance skills.
	“The rewarding music brings in enjoyment in the player.”	*“It is a difficult exercise and patients intended to do a wrong performance.”*	Patients should wear relatively tight clothes to ensure more accurate movement recognition by Kinect v2.
		“I was disappointed that the game did not recognize my squats while I was wearing my wide blouse.”	
Picking apples	“It is a nice game.”	“ *The supporting chairs interfere with Kinect’s recognition of the movements.”*	The chairs interfere with tracking for lunges.
		“The game does not respond to my correct movements.”	It is more suitable for patients who are at a later stage of their rehabilitation, that is, patients who can do lunges without balance support.
Football playing	“It is a very useful game for balance training, and it reacts very well to the movements of the player.”	*“Patients intended to shoot the ball.”*	To reduce cognitive load, let the patient play the game by doing back kicks using their left and right legs alternatively.
	“It is a great game. It made me feel like that I was playing a real football game.”	“It is difficult for me to pay attention to the arrows indicating which leg I should use.”	Instructions should tell the patients that they do not have to shoot the ball.
Fishing	“It is a nice game. I had a feeling that I had a real therapy.”	“I focused on the timer, and the virtual environment was not noticeable for me.”	Different types/sizes of fish need to be created in the game.
	“This game would help a lot in my rehabilitation.”	“All the fishes looked similar. It would be nice if I could catch a different fish.”	
	“The virtual environment is beautiful and I like it a lot!”		
General comments	*“Playing games distracted patients’ attention from exercise and pain.”*	*“Some patients were unsure of what action was supposed to take place at a particular time. When the patients fail in doing the exercise successfully, they do not get feedback on how to do it correctly.”*	*Real-time feedbacks on how far the patients are away from the required range of motion should be provided on the screen when the patients are performing the exercises.*
	*“You can use the game for fun besides the physiotherapy.”*	“You have to be clever enough to play the games as it requires paying attention to multiple things at the same time.”	To satisfy personal preferences, interfaces and virtual environments should be configurable to meet the needs of different cognitive challenges.
	“ *Patients have enjoyment and they can exercise by their own quickly by using the exercise games.”*	“I am very smart, so the game could be made slightly more difficult for me.”	
	“These are very nice and useful games and I would like to play them again.”		
	“You could play the game at home, but you would still need the physiotherapists’ feedback on how well you are doing with your rehabilitation by using the games.”		
	“It is good to receive feedback on the exercises from the games. It prevents you from doing the exercises in the wrong way.”		
	“After you get used to playing the exercise games, you have a lot of fun.”		

## Discussion

### Principal Findings

This study assessed the usability of the exercise games in terms of the experienced level of presence, enjoyment, exertion, pain, and technology acceptance among patients, and game design and technology acceptance among physiotherapists. The results showed that, in general, the patients experienced a high level of enjoyment, a moderate to high level of presence, and a low to moderate level of exertion and pain. The physiotherapists rated the exercise games as highly satisfying the nature of a motor rehabilitation program for elderly patients after hip surgery. Finally, both the patients and the therapists found the exercise games useful and easy to use and intended to use the exercise game system in the future.

The results of the evaluation of the game design are encouraging. The physiotherapists found all the exercise games meet the requirements for rehabilitation exercises [49]. The exercise games had a high level of adaptability to the patients’ motor skills, which is in line with the evenly distributed exertion levels experienced by the patients. The games were beneficial to the patients’ daily life activities such as walking, sitting, and standing. The games provided appropriate feedback on whether the patients exercise correctly and provided encouragement to the patients to continue with the therapy. The required range of motion such as step width, knee angle, and hip angle can be configured properly in the games. Furthermore, patients had high levels of enjoyment while exercising in the game. Therefore, the results suggest that the physiotherapists can devise novel rehabilitation programs by using our exercise games.

Patients experienced moderate to high levels of presence during the experiment with the lowest level of presence while playing the dancing game. During the dancing game, because of impaired balance skills, the patients used chairs to prevent fall. Hence, their attention was divided between the virtual and the real environment [52], which might explain the low level of presence in dancing. However, patients had quite a lot of enjoyment and little pain. Presence has been found to be associated with enjoyment. Earlier studies have found that participants experiencing high levels of enjoyment also show high levels of presence [53,54]. Being engaged in playing the game can also cause a decreased perception of pain [55]. Therefore, we expect that patients who can play the games without the support chair will experience a higher level of presence and enjoyment and less pain. In our experiment, all the patients used chairs as support.

According to the qualitative feedback, both the patients and the physiotherapists found squats the most difficult. Patients also reported the lowest enjoyment but highest exertion while doing squats in ringing the bell game. In an exercise game named Astrojumper, Finkelstein et al [56] found that participants’ ratings of perceived exertion positively correlated with their level of motivation. However, Rhodes et al [57] suggested that activities based on relatively moderate to low exertion and maximum enjoyment should be provided to increase adherence for elderly adults. Therefore, we suggest that to motivate the patients to use the game, squats should be used for patients who have relatively better balance skills.

Patients scored high on the technology acceptance scale, which was comparable with the scores in Wuest et al [32]. Patients found the exercise games understandable and easy to use. They found the exercises meaningful and useful for balance training. All the patients showed positive attitudes toward using the game in the rehabilitation center or at homes. Patients also expressed that they would like to continue to use the exercise games in their rehabilitation routinely. Our patients showed high acceptance of exercise games that were designed according to their cognitive and physical limitations. This finding is in line with the high acceptance rating of games that were specifically designed for patients, such as exercise games for home-based stroke rehabilitation [30,32].

In general, people are more inclined to use a system if they perceive it as useful, easy to use, and enjoyable [30]. Furthermore, earlier studies found that elderly participants strongly preferred virtual exercise gaming to traditional physical exercises [58,59]. Similarly, our system is designed to elicit increased motivation for rehabilitation, and the participants showed positive attitudes with regard to their gaming experience and the usability of the game system. Hence, we expect that the adherence rate of using our exercise games is higher than the traditional exercises, and participants will continue using our games when this system is implemented in the rehabilitation center or at homes.

### Limitations and Future Work

Apart from the contributions, there are still a number of limitations to this study. First, this study recruited a small sample of patients and physiotherapists because of limited availability of participants. Usability test with a larger group of elderly patients would be beneficial and allow exploration of usability within different subgroups, for example, patients who are at different recovery phases. It has been shown that with a pilot study of 4 or 5 participants, it is already possible to find 80% of the usability problems [60,61]. There were also 2 physiotherapists who got involved in the design of the exercise games, and 2 other physiotherapists evaluated the exercise games, which gives us confidence about the usability about our system at the level of therapists.

Second, most of the exercises had to be performed while holding onto a chair, which sometimes influenced the tracking accuracy for exercises such as squats and lunges. Similarly, Ofli et al [62] reported that the highest tracking errors were found in hip and ankle joints while using Kinect. Hence, we suggest that patients who are at the beginning phase of physiotherapy after hip surgery should use the exercise games such as cycling, dancing, football, and fishing, which are not affected by supporting objects. Furthermore, we did meet problems with Kinect v2 recognizing obese patients for playing apple picking lunges. Patients with obesity may not be recognized as correctly as patients with an average body mass [63]. Future research should look into the difference in tracking accuracy and reliability comparing people with different levels of body mass.

Third, some user aspects of the game design such as high cognitive load and lack of real-time feedback on wrong movements and mismatched movements would pose barriers to future use. To address these issues, we plan to include the customization of the user interface and virtual environment according to personal preferences for cognitive challenges by providing real-time feedback on how far the patients are away from the required range of motion when they are performing the exercises and by giving informative instructions at the beginning of each exercise game. Furthermore, there are still some minor problems with the games that need to be fixed; for example, the setting of going to the same direction for more than once in dancing should be configurable in the therapist interface.

Finally, in this pilot test, we focused on the usability of the exercise games. However, it would be interesting to assess the usability of the CC360 and find ways to improve it according to elderly people’s abilities and preferences when the system is ready in the rehabilitation center or at homes.

The social aspect is known to affect exercise adherence [62]. In general, social support can increase self-efficacy and then enhance adherence [64]. In this study, as a first step, the exercise games will be used in the rehabilitation center, where different patients exercise in a common area. Thus, the patients can share their scores with others, forming a healthy competitive exercise environment among the elderly patients. Future work such as creating leader boards and score-based achievements will also help foster competition with the possibility of cooperation among patients and enabling social engagement during the exercise. Furthermore, CC360 provides accomplished exercise record of patients. In this way, the patients can be monitored by physiotherapists and other caregivers and encouraged to adhere to the exercises by playing the game.

As a next step, the effectiveness of the exercise games will be tested in a randomized controlled trial with 15 patients in each group, that is, an experimental group combining traditional exercises with playing exercise games versus control group with traditional exercises. The study will be conducted in the rehabilitation center and can shed some light on how motivating the game system is over a period of time.

### Conclusions

We created Fietsgame, an engaging and motivating exercise game system, which translates traditional rehabilitation exercises into playful exercises. The performance of the users was automatically tracked using a 3D depth camera and stored for further analysis by the physiotherapists. The results indicate that the game can be used by patients as a new rehabilitation tool after hip surgery, and both the patients and the physiotherapists expressed positive attitudes toward using the game in the future. Although this study had a limited number of participants, it provides sufficient insights on the usability of the system and suggests improvements in the future. The qualitative feedback revealed that exercise games designed for elderly patients should be challenging enough to keep their interest and attention, but also should take into account their impaired motor, sensory, and cognition functions. We will improve the game by including real-time corrective feedback when patients are performing the exercises, by providing a customizable user interface allowing adjustments to cognitive load and by creating more varieties of game content. A randomized controlled clinical trial will be conducted covering a longer time period, testing the effectiveness of the game. The final goal is to provide elderly patients with a game that can be used in nursery houses or at homes to achieve improved physical functions and maintain independent living.

## Appendix

Multimedia Appendix 1 The original physiotherapist control interface of CC360.

Multimedia Appendix 2 The original patient interface showing the patient’s medical record in CC360.

Multimedia Appendix 3 Questionnaires answered by the patients.

Multimedia Appendix 4 Questionnaires answered by the physiotherapists.

## Abbreviations

CC360: Community Care 360
CGI: Consultants to Government and Industries
IoT: Internet of Things
IPQ: Igroup presence questionnaire
PC: personal computer
SD: standard deviation
TAM: Technology Acceptance Model
VAS: Visual Analogue Score
