# Supplementary figures and images for: Usability Test of Exercise Games Designed for Rehabilitation of Elderly Patients After Hip Replacement Surgery: Pilot Study
Source: JMIR Serious Games. 2017 Oct 12;5(4):e19. doi: 10.2196/games.7969 (PMC5658642; doi:10.2196/games.7969)

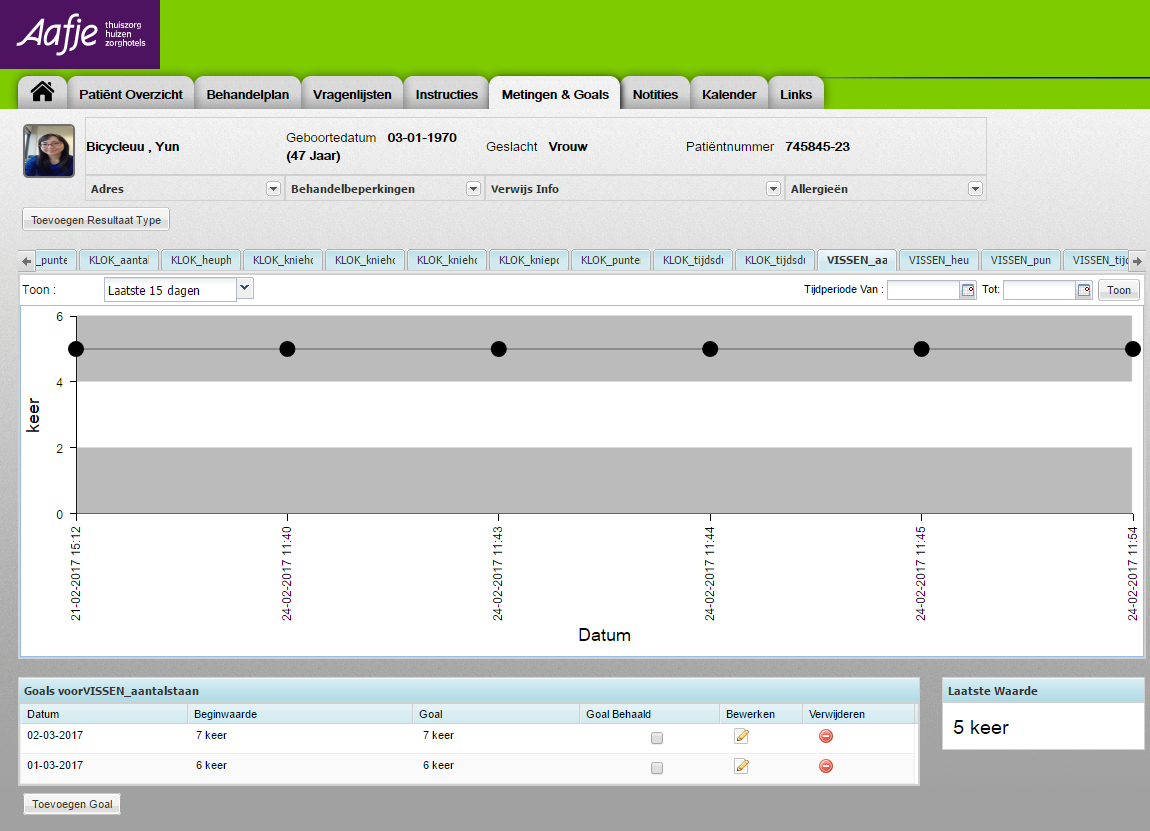

Supplement: Multimedia Appendix 1 [file games_v5i4e19_app1.png]

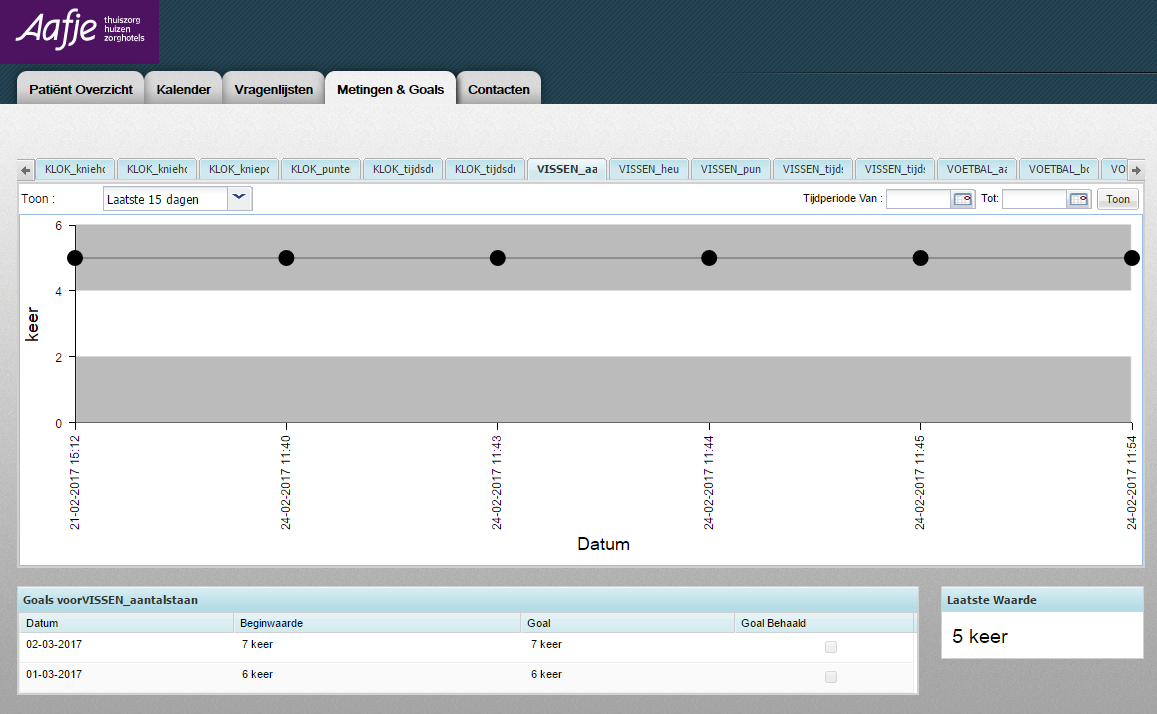

Supplement: Multimedia Appendix 2 [file games_v5i4e19_app2.png]
