# Supplementary material for: Usability Test of Exercise Games Designed for Rehabilitation of Elderly Patients After Hip Replacement Surgery: Pilot Study
Source: JMIR Serious Games. 2017 Oct 12;5(4):e19. doi: 10.2196/games.7969 (PMC5658642; doi:10.2196/games.7969)
Supplement: Multimedia Appendix 3 [file games_v5i4e19_app3.pdf]

# Questionnaires for the usability test of the Fietsgame

## Part one: basic demographic information

Before you start the exercise game, please fill in your information below.

Participant number: to be filled in by the experimenter (___)___

Date of intake:_________________

Number of exercise sessions per day:_______________________

Age:____________

Gender: __________

Language:_______________________________________

What is your highest level of education so far?________________________________________

Visual acuity, left eye______

Right eye_____

What is your sport habits?______________________________________________________

How often do you play computer games?

- Never
- Occasionally (once or twice per month)
- Often but less than 50% of days
- 50 or more of days
- Everyday

Do you have photosensitive epilepsy?

- Yes
- No

Do you live alone or with someone else?

- I live alone
- I live with______________________________

## Part two: gaming experience

Game:_____________

Now you'll see some statements about experiences. Please indicate, whether or not each statement applies to your experience. You can use the whole range of answers. There are no right or wrong answers, only your opinion counts. Please answer all these questions only referring to the last exercise game you experienced.

## Presence

In the computer generated world I had a sense of "being there".

| | | | | | | | | |
|---|---|---|---|---|---|---|---|---|
| not at all | ○ | ○ | ○ | ○ | ○ | ○ | ○ | very much |
| | -3 | -2 | -1 | 0 | +1 | +2 | +3 | |

## Enjoyment Scale

Do you find the exercise game interesting?

| | | | | | | | | |
|---|---|---|---|---|---|---|---|---|
| I dislike it | ○ | ○ | ○ | ○ | ○ | ○ | ○ | I like it |
| | -3 | -2 | -1 | 0 | +1 | +2 | +3 | |

## Perceived exertion

Please rate your perceived physical exertion during the game:

| | |
|---|---|
| 6 | |
| 7 | Very, very light |
| 8 | |
| 9 | Very light |
| 10 | |
| 11 | Fairly light |
| 12 | |
| 13 | Somewhat hard |
| 14 | |
| 15 | Hard |
| 16 | |
| 17 | Very hard |
| 18 | |
| 19 | Very, very hard |
| 20 | |

## Visual Analog Scale (pain scale)

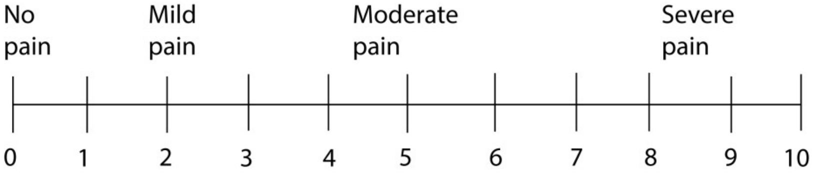


## Questions or comments

Do you have any questions or comments on this exercise game?

# Part three: Technology acceptance model

Please indicate, whether or not each statement applies to your experience. You can use the whole range of answers. There are no right or wrong answers, only your opinion counts. You will notice that some questions are very similar to each other. This is necessary for statistical reasons. Please answer all these questions only referring to all the exercise games you experienced.

### Perceived usefulness

Using exercise game can enable me to complete rehabilitation more quickly.

strongly disagree ○ ○ ○ ○ ○ ○ ○ strongly agree

-3 -2 -1 0 +1 +2 +3

Using exercise game CANNOT improve my rehabilitation.

strongly disagree ○ ○ ○ ○ ○ ○ ○ strongly agree

-3 -2 -1 0 +1 +2 +3

Using exercise game can increase my productivity in rehabilitation.

strongly disagree ○ ○ ○ ○ ○ ○ ○ strongly agree

-3 -2 -1 0 +1 +2 +3

Using exercise game CANNOT enhance my rehabilitation effectiveness.

| strongly disagree | ○ | ○ | ○ | ○ | ○ | ○ | ○ | strongly agree |
|---|---|---|---|---|---|---|---|---|
| | -3 | -2 | -1 | 0 | +1 | +2 | +3 | |

Using exercise game can make my rehabilitation easier.

| strongly disagree | ○ | ○ | ○ | ○ | ○ | ○ | ○ | strongly agree |
|---|---|---|---|---|---|---|---|---|
| | -3 | -2 | -1 | 0 | +1 | +2 | +3 | |

I would find exercise game NOT useful for my rehabilitation.

| strongly disagree | ○ | ○ | ○ | ○ | ○ | ○ | ○ | strongly agree |
|---|---|---|---|---|---|---|---|---|
| | -3 | -2 | -1 | 0 | +1 | +2 | +3 | |

## Perceived ease of use

Learning to operate exercise game would NOT be easy for me.

| strongly disagree | ○ | ○ | ○ | ○ | ○ | ○ | ○ | strongly agree |
|---|---|---|---|---|---|---|---|---|
| | -3 | -2 | -1 | 0 | +1 | +2 | +3 | |

I would find it easy to get exercise game to do what I need to do in my rehabilitation.

| strongly disagree | ○ | ○ | ○ | ○ | ○ | ○ | ○ | strongly agree |
|---|---|---|---|---|---|---|---|---|
| | -3 | -2 | -1 | 0 | +1 | +2 | +3 | |

My interaction with exercise game would be clear and understandable.

| strongly disagree | ○ | ○ | ○ | ○ | ○ | ○ | ○ | strongly agree |
|---|---|---|---|---|---|---|---|---|
| | -3 | -2 | -1 | 0 | +1 | +2 | +3 | |

I find exercise game INFLEXIBLE to interact with.

strongly disagree ○ ○ ○ ○ ○ ○ ○ strongly agree

-3 -2 -1 0 +1 +2 +3

It is NOT easy for me to become skillful in using exercise game.

strongly disagree ○ ○ ○ ○ ○ ○ ○ strongly agree

-3 -2 -1 0 +1 +2 +3

I would find exercise game easy to use.

strongly disagree ○ ○ ○ ○ ○ ○ ○ strongly agree

-3 -2 -1 0 +1 +2 +3

## Attitude

Using exercise game in rehabilitation is a good idea.

strongly disagree ○ ○ ○ ○ ○ ○ ○ strongly agree

-3 -2 -1 0 +1 +2 +3

Using exercise game in rehabilitation is UNPLEASANT.

strongly disagree ○ ○ ○ ○ ○ ○ ○ strongly agree

-3 -2 -1 0 +1 +2 +3

Using exercise game is beneficial for my rehabilitation.

strongly disagree ○ ○ ○ ○ ○ ○ ○ strongly agree

-3 -2 -1 0 +1 +2 +3

## Intention to use

I intend to use exercise game in my rehabilitation when it becomes available in my home.

| strongly disagree | -3 | -2 | -1 | 0 | +1 | +2 | +3 | strongly agree |
|---|---|---|---|---|---|---|---|---|

I intend to use exercise game as often as needed.

| strongly disagree | -3 | -2 | -1 | 0 | +1 | +2 | +3 | strongly agree |
|---|---|---|---|---|---|---|---|---|

I intend NOT to use exercise game in rehabilitation routinely.

| strongly disagree | -3 | -2 | -1 | 0 | +1 | +2 | +3 | strongly agree |
|---|---|---|---|---|---|---|---|---|

Whenever possible, I intend NOT to use exercise game in rehabilitation.

| strongly disagree | -3 | -2 | -1 | 0 | +1 | +2 | +3 | strongly agree |
|---|---|---|---|---|---|---|---|---|

To the extent possible, I would use exercise game to do different things, clinical or nonclinical.

| strongly disagree | -3 | -2 | -1 | 0 | +1 | +2 | +3 | strongly agree |
|---|---|---|---|---|---|---|---|---|

To the extent possible, I would use exercise game in rehabilitation frequently.

| strongly disagree | -3 | -2 | -1 | 0 | +1 | +2 | +3 | strongly agree |
|---|---|---|---|---|---|---|---|---|

## Final questions

In general, what did you like about your session on the exercise game?

In general, what did you not like about your session on the exercise game?
