# Supplementary material for: Usability Test of Exercise Games Designed for Rehabilitation of Elderly Patients After Hip Replacement Surgery: Pilot Study
Source: JMIR Serious Games. 2017 Oct 12;5(4):e19. doi: 10.2196/games.7969 (PMC5658642; doi:10.2196/games.7969)
Supplement: Multimedia Appendix 4 [file games_v5i4e19_app4.pdf]

Questionnaires for the usability test of the Fietsgame

Part one: basic demographic information

Before you start the exercise game, please fill in your information below.

Participant number: please do not fill in (____)

Age:____________

Gender: __________

Nationality:____________

Language:_______________________________________

What is your highest level of education so far?____

How often do you play computer games?

- Never
- Occasionally (once or twice per month)
- Often but less than 50% of days
- 50 or more of days
- Everyday

Do you have photosensitive epilepsy?

- Yes
- No

Part two: gaming experience

Game:____________

Now you'll see some statements about experiences. Please indicate, whether or not each statement applies to your experience. You can use the whole range of answers. There are no right or wrong answers, only your opinion counts. Please answer all these questions only referring to the LAST VIRTUAL REALITY you experienced.

Criteria for rehabilitation

Please rate whether the games satisfy the nature of a motor rehabilitation program.

Having adaptability to motor skill level (changeable level of difficulty to promote improvement)

strongly disagree ○ ○ ○ ○ ○ ○ ○ strongly agree

-3 -2 -1 0 +1 +2 +3

Having meaningful tasks (its correlation with daily living)

strongly disagree ○ ○ ○ ○ ○ ○ ○ strongly agree

-3 -2 -1 0 +1 +2 +3

Having appropriate feedback

strongly disagree ○ ○ ○ ○ ○ ○ ○ strongly agree

-3 -2 -1 0 +1 +2 +3

In therapy appropriate range of motion (quality of the exercise for treatment purpose)

strongly disagree ○ ○ ○ ○ ○ ○ ○ strongly agree

-3 -2 -1 0 +1 +2 +3

Focus diverted from exercise (enjoyment of game play etc.)

strongly disagree ○ ○ ○ ○ ○ ○ ○ strongly agree

-3 -2 -1 0 +1 +2 +3

Questions or comments

Do you have any questions or comments on this exercise game?

Part three: Technology acceptance model

Please indicate, whether or not each statement applies to your experience. You can use the whole range of answers. There are no right or wrong answers, only your opinion counts. You will notice that some questions are very similar to each other. This is necessary for statistical reasons. Please answer all these questions only referring to all the exercise games you experienced.

Perceived usefulness

Using exercise game can enable me to complete patient care more quickly.

strongly disagree ○ ○ ○ ○ ○ ○ ○ strongly agree

-3 -2 -1 0 +1 +2 +3

Using exercise game CANNOT improve my patient care and management.

strongly disagree ○ ○ ○ ○ ○ ○ ○ strongly agree

-3 -2 -1 0 +1 +2 +3

Using exercise game can increase my productivity in patient care.

strongly disagree ○ ○ ○ ○ ○ ○ ○ strongly agree

-3 -2 -1 0 +1 +2 +3

Using exercise game CANNOT enhance my service effectiveness.

strongly disagree ○ ○ ○ ○ ○ ○ ○ strongly agree

-3 -2 -1 0 +1 +2 +3

Using exercise game can make my patient care and management easier.

strongly disagree ◯ ◯ ◯ ◯ ◯ ◯ ◯ strongly agree

-3 -2 -1 0 +1 +2 +3

I would find exercise game NOT useful for my patient care and management.

strongly disagree ◯ ◯ ◯ ◯ ◯ ◯ ◯ strongly agree

-3 -2 -1 0 +1 +2 +3

Perceived ease of use

Learning to operate exercise game would NOT be easy for me.

strongly disagree ◯ ◯ ◯ ◯ ◯ ◯ ◯ strongly agree

-3 -2 -1 0 +1 +2 +3

I would find it easy to get exercise game to do what I need to do in my patient care and management.

strongly disagree ◯ ◯ ◯ ◯ ◯ ◯ ◯ strongly agree

-3 -2 -1 0 +1 +2 +3

My interaction with exercise game would be clear and understandable.

strongly disagree ◯ ◯ ◯ ◯ ◯ ◯ ◯ strongly agree

-3 -2 -1 0 +1 +2 +3

I find exercise game INFLEXIBLE to interact with.

strongly disagree ◯ ◯ ◯ ◯ ◯ ◯ ◯ strongly agree

-3 -2 -1 0 +1 +2 +3

It is NOT easy for me to become skillful in using exercise game.

strongly disagree ○ ○ ○ ○ ○ ○ ○ strongly agree

-3 -2 -1 0 +1 +2 +3

I would find exercise game easy to use.

strongly disagree ○ ○ ○ ○ ○ ○ ○ strongly agree

-3 -2 -1 0 +1 +2 +3

Attitude

Using exercise game in patient care and management is a good idea.

strongly disagree ○ ○ ○ ○ ○ ○ ○ strongly agree

-3 -2 -1 0 +1 +2 +3

Using exercise game in patient care and management is UNPLEASANT.

strongly disagree ○ ○ ○ ○ ○ ○ ○ strongly agree

-3 -2 -1 0 +1 +2 +3

Using exercise game is beneficial to my patient care and management.

strongly disagree ○ ○ ○ ○ ○ ○ ○ strongly agree

-3 -2 -1 0 +1 +2 +3

Intention to use

I intend to use exercise game in my patient care and management when it becomes available in my

department or hospital.

strongly disagree	○	○	○	○	○	○	○	strongly agree
	-3	-2	-1	0	+1	+2	+3	

I intend to use exercise game to provide health-care services to patients as often as needed.

strongly disagree	○	○	○	○	○	○	○	strongly agree
	-3	-2	-1	0	+1	+2	+3	

I intend NOT to use exercise game in my patient care and management routinely.

strongly disagree	○	○	○	○	○	○	○	strongly agree
	-3	-2	-1	0	+1	+2	+3	

Whenever possible, I intend NOT to use exercise game in my patient care and management.

strongly disagree	○	○	○	○	○	○	○	strongly agree
	-3	-2	-1	0	+1	+2	+3	

To the extent possible, I would use exercise game to do different things, clinical or nonclinical.

strongly disagree	○	○	○	○	○	○	○	strongly agree
	-3	-2	-1	0	+1	+2	+3	

To the extent possible, I would use exercise game in my patient care and management frequently.

strongly disagree	○	○	○	○	○	○	○	strongly agree
	-3	-2	-1	0	+1	+2	+3	

Final questions

For the therapist interface, what kind of information are you still missing?

In general, what did you like about your session on the exercise game?

In general, what did you not like about your session on the exercise game?
